# Factoring-in agglomeration of carbon nanotubes and nanofibers for better prediction of their toxicity versus asbestos

**DOI:** 10.1186/1743-8977-9-10

**Published:** 2012-04-10

**Authors:** Ashley R Murray, Elena R Kisin, Alexey V Tkach, Naveena Yanamala, Robert Mercer, Shih-Houng Young, Bengt Fadeel, Valerian E Kagan, Anna A Shvedova

**Affiliations:** 1 Pathology and Physiology Research Branch, Health Effects Laboratory Division, National Institute for Occupational Safety and Health, Morgantown, WV, USA; 2 Department of Physiology and Pharmacology, West Virginia University, Morgantown, WV, USA; 3 Department of Environmental and Occupational Health, University of Pittsburgh, Pittsburgh, PA, USA; 4 Department of Structural Biology, University of Pittsburgh, Pittsburgh, PA, USA; 5 Division of Molecular Toxicology, Institute of Environmental Medicine, Karolinska Institutet, Stockholm, Sweden; 6 Health Effects Laboratory Division, Pathology and Physiology Research Branch, NIOSH, M/L 2015, 1095 Willowdale Road, Morgantown, WV 26505, USA

## Abstract

**Background:**

Carbon nanotubes (CNT) and carbon nanofibers (CNF) are allotropes of carbon featuring fibrous morphology. The dimensions and high aspect ratio of CNT and CNF have prompted the comparison with naturally occurring asbestos fibers which are known to be extremely pathogenic. While the toxicity and hazardous outcomes elicited by airborne exposure to single-walled CNT or asbestos have been widely reported, very limited data are currently available describing adverse effects of respirable CNF.

**Results:**

Here, we assessed pulmonary inflammation, fibrosis, oxidative stress markers and systemic immune responses to respirable CNF in comparison to single-walled CNT (SWCNT) and asbestos. Pulmonary inflammatory and fibrogenic responses to CNF, SWCNT and asbestos varied depending upon the agglomeration state of the particles/fibers. Foci of granulomatous lesions and collagen deposition were associated with dense particle-like SWCNT agglomerates, while no granuloma formation was found following exposure to fiber-like CNF or asbestos. The average thickness of the alveolar connective tissue - a marker of interstitial fibrosis - was increased 28 days post SWCNT, CNF or asbestos exposure. Exposure to SWCNT, CNF or asbestos resulted in oxidative stress evidenced by accumulations of 4-HNE and carbonylated proteins in the lung tissues. Additionally, local inflammatory and fibrogenic responses were accompanied by modified systemic immunity, as documented by decreased proliferation of splenic T cells *ex vivo *on day 28 post exposure. The accuracies of assessments of effective surface area for asbestos, SWCNT and CNF (based on geometrical analysis of their agglomeration) versus estimates of mass dose and number of particles were compared as predictors of toxicological outcomes.

**Conclusions:**

We provide evidence that effective surface area along with mass dose rather than specific surface area or particle number are significantly correlated with toxicological responses to carbonaceous fibrous nanoparticles. Therefore, they could be useful dose metrics for risk assessment and management.

## Background

Carbon nanotubes (CNT), including single-walled (SWCNT), double-, and multi-walled (MWCNT), and carbon nanofibers (CNF) are allotropes of carbon featuring fibrous morphology. SWCNT (typically 0.4 - 3 nm in diameter) are composed of a single cylindrical sheet of graphene, MWCNT (2-200 nm in diameter) consist of several concentric, coaxial rolled up graphene sheets [1,2]. In contrast to CNT, CNF represent a less perfect graphene sheet arrangement, with layers of graphene stacked at an angle to the fiber axis. CNF, formed from graphene nanocones or "cups" and sometimes referred to as "stacked-cup carbon nanotubes", are strong flexible filaments ranging from 70-200 nm in diameter and 10 μm - 100 μm in length. They are advantageous for a broad variety of applications, such as nanocomposites and biomedical devices. Due to their cost-effectiveness, the commercial use of CNF has grown exponentially [3,4]. CNT and CNF are elongated structures with a high aspect ratio (up to 1:1000, length/width) produced predominantly by HiPco, chemical vapor deposition, laser ablation or arc discharge by employing a variety of catalytic metal particles [5]. The purity of CNT and CNF depends on the technology used and on the subsequently applied purification procedures. There is considerable debate on how morphology, physical and chemical properties, including size, shape, charge and/or agglomeration state, are translated to the toxicity of fibrous nanomaterials (NM). The dimensions and high aspect ratio of CNT and CNF have driven their comparisons with naturally occurring asbestos fibers, which are known to be extremely pathogenic [6-8]. However, while the toxicity and hazardous outcomes elicited by airborne exposure to SWCNT and MWCNT has been widely reported [9-15], very limited data are currently available describing the adverse effects of respirable CNF.

Physical dimensions, surface properties and biopersistence are key factors underlying the potential toxicity of fibrous NM. Their aerodynamic diameter along with particle dimensions dictates the pattern of deposition within lungs [16,17]. The primary site for deposition of fibrous NP is the alveolar region. In the alveolar area, NP are phagocytosed by macrophages (MΦ) and cleared via the mucociliary escalator. While short fibers are efficiently cleared, the longer ones are retained in the lung thus causing a persistent lung burden [18,19]. Phagocytosis targets fibrous NP aiming to break them down. However, many fibrous NP are chemically resistant and cannot be readily dissolved in physiological conditions. Repeated attempts to phagocytose asbestos fibers by MΦ trigger a cascade of amplifying events resulting in the generation of reactive oxygen/nitrogen species (ROS/NOS), release of inflammatory cytokines and/or chemokines, and leaking of lysosomal enzymes, thus leading to cellular injury and pulmonary inflammation frequently culminating with acute pneumonia [18].

Exposure to amphibole or crocidolite asbestos environmentally and/or in the workplace has been strongly associated with pulmonary fibrosis, autoimmune diseases and mesothelioma [20]. In light of recently reported genotoxic effects resembling those seen after asbestos exposure, there is a huge debate regarding whether exposure to fibrous carbon-derived nanomaterials (NM) follow a similar paradigm [4,21-23].

Because the effects of airborne CNF have not been previously addressed, we designed a comparative study assessing pulmonary inflammation, fibrosis, and systemic immune responses to respirable CNF, SWCNT, and asbestos. Pulmonary outcomes along with innate and systemic immune responses were evaluated at 1, 7, and 28 days post exposure. The obtained data provide evidence that respirable CNF are quite hazardous, exhibiting similar pulmonary responses to those seen following SWCNT and asbestos exposure. While acute pulmonary inflammation and fibrosis induced by CNF and asbestos were delayed as compared to SWCNT, the systemic immune response elicited by CNF was akin to that observed for asbestos. Exposure to CNF and SWCNT was found to facilitate persistent pulmonary fibrosis along with immune suppression resembling the effects of asbestos, which could potentially promote progression of neoplastic lesions and cancer.

## Results

### Particle characterization

**Figure 1:**
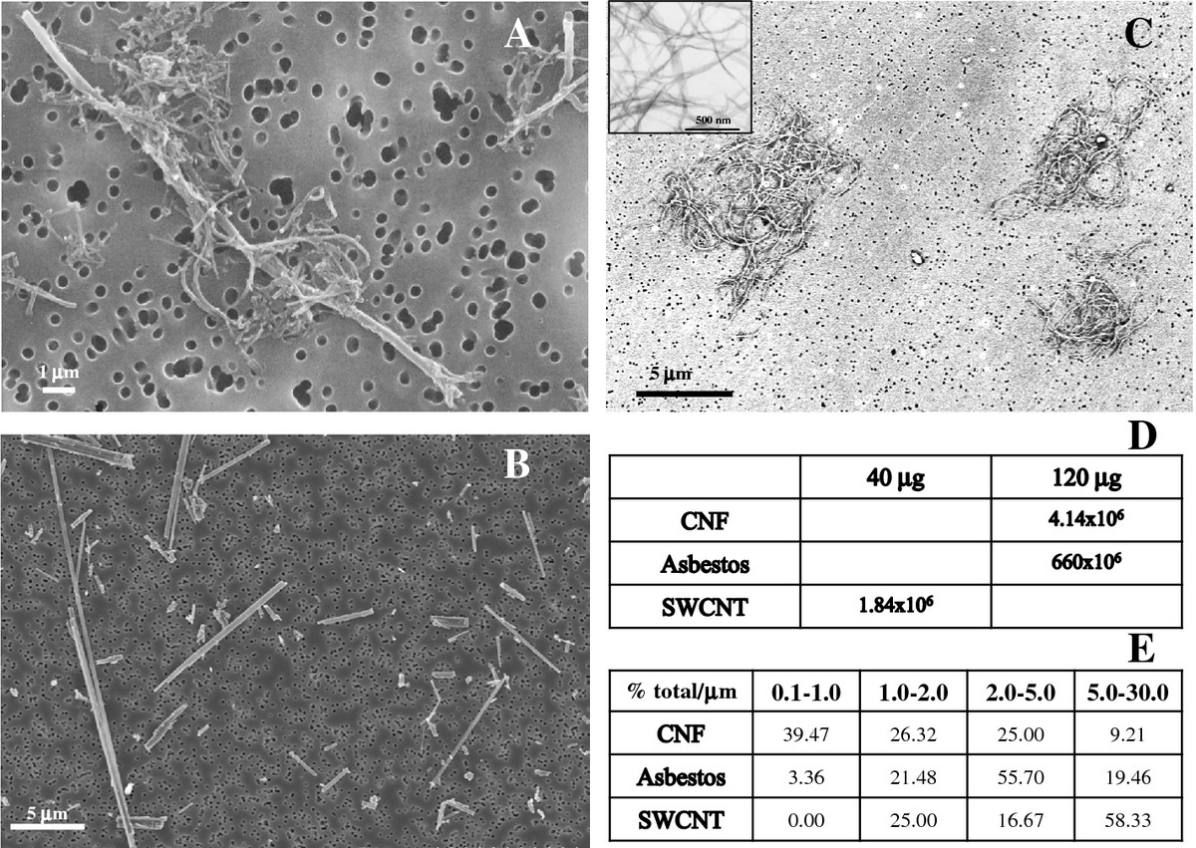
**Scanning Electron Microscopy images of CNF (A), asbestos (B), SWCNT (C, Inset: TEM showing individual roped SWCNT), structure numbers per dose (D) and particles size (length) distribution (E) presented as % of total particles**.

All particles utilized in the current study were characterized by chemical analysis by NMAM #5040 and ICP-AES. Pyrograf CNF was found to be 98.6% wt. elemental carbon with iron levels of 1.4% wt. CNF diameters ranged from 80 to 160 nm. A specific surface area (SSA) of CNF was 35-45 m^2^/g (measured by BET). Length was determined by SEM and found to be approximately 5-30 μm (Figure 1). SWCNT were 99.7% wt. elemental carbon with 0.23% wt iron. Individual SWCNT had diameters ranging from 1 to 4 nm and were 1-3 μm in length. SWCNT were found to have a specific surface area of 1040 m^2^/g. As evidenced by TEM, individual SWCNT were bundled in ropes with diameters of ~65 nm (Figure 1C, inset). Crocidolite asbestos fibers lengths were within 2-30 μm range and width of 160-800 nm. Asbestos had a surface area of 8.3 m^2^/g. Iron levels in crocidolite asbestos were found to be 18% wt. In the suspensions of asbestos and CNF utilized in the current study, a substantial amount of particles/bundles had a conventional fibrous morphology falling under WHO definition: e.g. length > 5 micrometer, diameter < 3 micrometer (Figure 1E). Individual SWCNT bundles in our preparations also fit into the fiber definition; however, they were agglomerated into tertiary structures, which, of course, no longer possess a fibrous morphology (Figure 1C).

### Calculations of effective surface area of SWCNT and CNF using geometrical analysis

**Figure 2:**
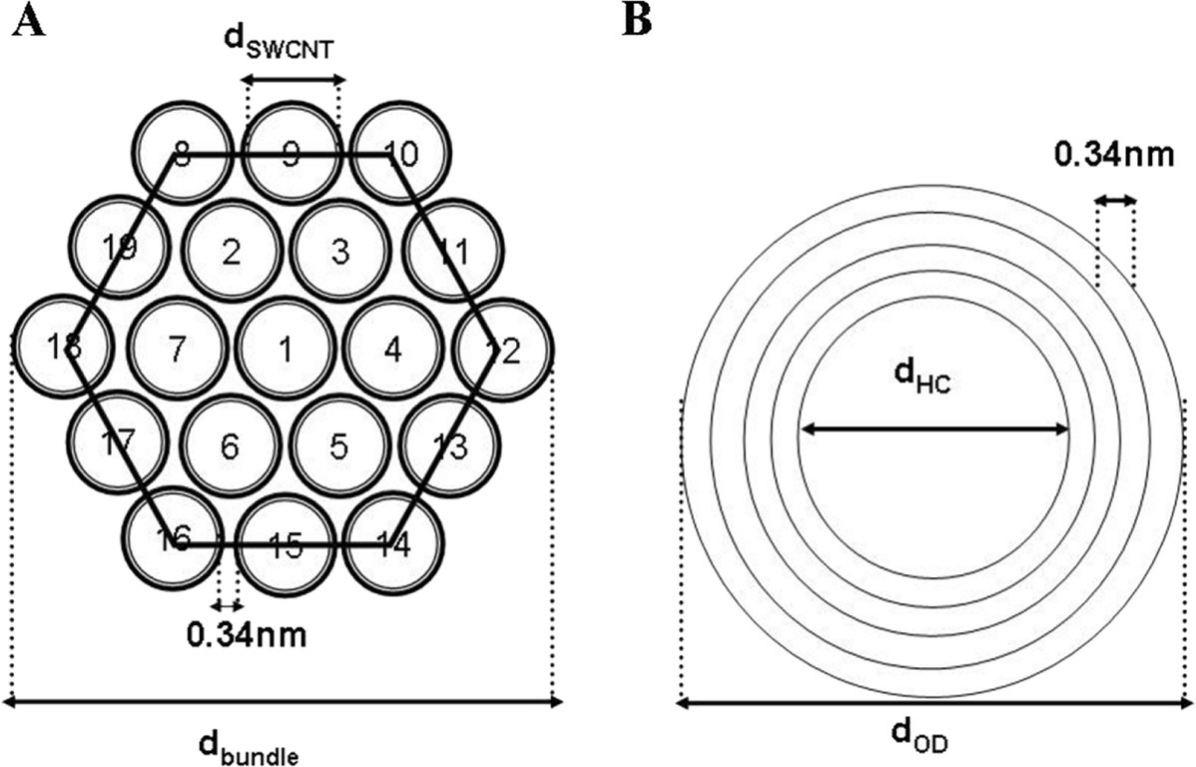
**Schematic representation of the SWCNT bundles and CNF**. (A) A hexagonal arrangement model of a bundle containing two layers of SWCNT. The labels d_SWCNT _and d_bundle _correspond to the diameters of SWCNT and a bundle of SWCNT, respectively. (B) A model of CNF showing five carbon layers. The labels d_OD_, and d_HC _correspond to the outer and hollow core diameters of CNF, respectively.

Geometrical analysis of the surface areas of SWCNT and CNF agglomerates were conducted as follows. The characterization of SWCNT particles using TEM indicated that a bundled rope of SWCNT (Figure 1C, inset) has an average diameter of ~65 nm. It should be noted that the manufacturer's specifications for SWCNT had a specific surface area (SSA) of 1040 m^2^/g and diameters ranging from 1 to 4 nm, significantly lower than what we observed. This apparent discrepancy in diameters may be due to the tendency of SWCNT to form bundles because of Van der Waals interactions. Hence, it becomes important to understand, how a bundle with N identical SWCNT arranged in the form of an agglomerated network (Figure 2A) affects the effective surface area. Assuming that each SWCNT has an average diameter of ~3 nm ((2 + 3 + 4)/3 = 3), we estimated that a SWCNT bundle of diameter ~65 nm will contain a total of ~295 SWCNT arranged in 10 layers with an effective accessible surface area equivalent to ~31 SWCNT (see Methods). Therefore, the effective surface area of SWCNT bundles can be estimated as 138.2 m^2^/g (using eq (4): 1315 × (31/295) m^2^/g). Thus, the effective surface area of SWCNT of diameter ~3 nm decreases from 1040 m^2^/g to 138 m^2^/g when it forms SWCNT bundles with a diameter of ~65 nm. Similarly, from the SEM images (Figure 1A), we determined that CNF particles had an average hollow core and outer diameters (Figure 2B) of ~53 nm and ~109 nm, respectively. Using equations 5 & 6 (see Methods), we estimated that CNF particles are made up of ~82 carbon layers, and have an effective surface area of ~21 m^2^/g. Based on these calculations, the effective surface area of 40 μg of SWCNT and 120 μg of CNF will correspond to 5.52 × 10^-3 ^m^2^/g and 2.52 × 10^-3 ^m^2^/g. As a result, the effective surface area of SWCNT and CNF administered is ~5.8 times and ~2.6 times higher, respectively as compared to asbestos (9.6 × 10^-4 ^m^2^/mouse).

### Characterization of pulmonary inflammatory response

**Figure 3:**
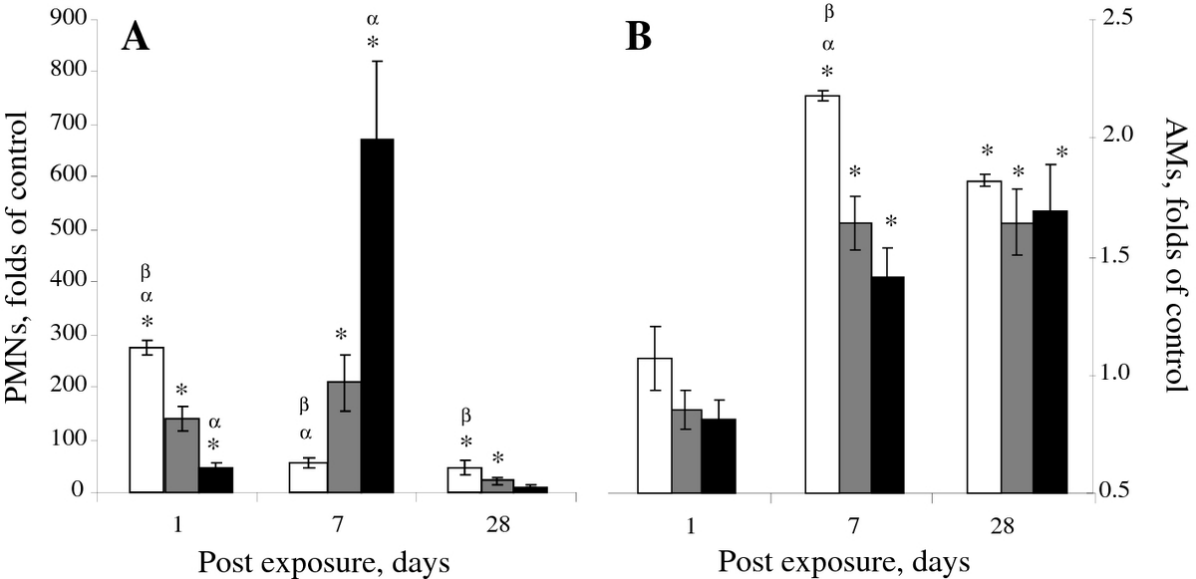
**Cell profile in BAL fluids of C57BL/6 mice after pharyngeal aspiration with CNF, Crocidolite Asbestos, or SWCNT**. A: polymorphonuclear leukocytes (PMNs); B: alveolar macrophages (AMs). Open columns - exposure with SWCNT (40 μg/mouse); gray columns - exposure with CNF (120 μg/mouse); black columns - exposure with crocidolite asbestos (120 μg/mouse). Mice were exposed via pharyngeal aspiration to doses indicated. Animals were sacrificed 1, 7, and 28 days post exposure. Average control (PBS-treated mice) values for PMNs (cells, x10^3^) on day 1, 7 or 28 post exposure were 1.58 ± 0.69, 1.39 ± 0.45 or 0.71 ± 0.24, respectively. Average control values for AMs (cells, x10^3^) on day 1, 7 or 28 post exposure were 389.06 ± 22.80, 345.01 ± 30.01 or 229.79 ± 27.81, respectively. Means ± SE (*n *= 6 mice per group). *p < 0.05 vs. control mice, ^α^p < 0.05 vs. mice exposed to CNF, ^β^p < 0.05 vs. mice exposed to asbestos.

In order to evaluate lung injury and inflammatory responses to CNF particles, comparing to those observed in SWCNT and asbestos, cell differential and total BAL cell counts, permeability of the lung epithelium (protein levels), and cell damage (LDH release) were determined 1, 7, and 28 days following pharyngeal aspiration of nanoparticles/fibers in C57BL/6 mice. Analysis of the pulmonary inflammatory response following CNF and SWCNT exposure indicated an accumulation of PMNs (150 and 700 fold vs control, respectively, Figure 3A) on day 1 followed by an influx of AMs (Figure 3B) peaking on day 7 (2.0 and 1.6 fold vs control, respectively). In comparison, exposure to asbestos induced a "delayed" inflammatory response with maximal PMN influx (675-fold vs control) occurring on day 7 post exposure. By day 28 post exposure to CNF, PMNs, and AMs in BAL fluid substantially decreased; however, the numbers still remained elevated (25-, and 1.6 fold, respectively) as compared to control.

**Figure 4:**
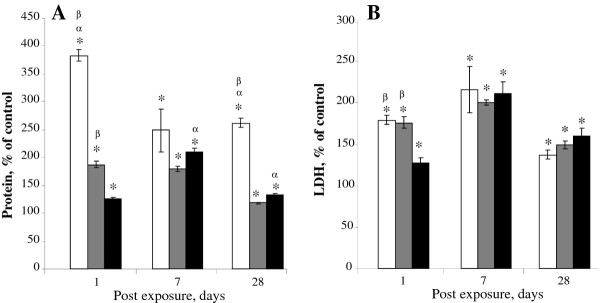
**Pulmonary cell damage as evaluated by changes in LDH activity and air/blood barrier damage was evaluated by protein in the bronchoalveloar lavage fluid of C57BL/6 mice in response aspiration of CNF, Crocidolite Asbestos or SWCNT**. A: protein; B: LDH. Open columns - exposure with SWCNT (40 μg/mouse); gray columns - exposure with CNF (120 μg/mouse); black columns - exposure with crocidolite asbestos (120 μg/mouse). Mice were exposed via pharyngeal aspiration to the doses indicated. Animals were sacrificed 1, 7, and 28 days post exposure. Average control (PBS-treated mice) values for protein (mg/ml) on day 1, 7 or 28 post exposure were 0.31 ± 0.05, 0.33 ± 0.05 or 0.38 ± 0.05, respectively. Average control values for LDH (U/ml) on day 1, 7 or 28 post exposure were 25.12 ± 0.70, 26.81 ± 1.03 or 25.47 ± 1.06, respectively. Means ± SE (*n *= 6 mice per group). *p < 0.05 vs. control mice, ^α^p < 0.05 vs. mice exposed to CNF, ^β^p < 0.05 vs. mice exposed to asbestos.

Exposure to SWCNT, CNF or asbestos caused increased lung permeability, as evidenced by elevated total protein in the BAL fluid. CNF exposure (120 μg/mouse) induced a 1.86-, 1.75-, and 1.14-fold increase in BAL protein on days 1, 7, and 28 post exposure, respectively (Figure 4A). In comparison, SWCNT (40 μg/mouse) exposure resulted in a raise in protein up to 3.75, 2.5 and 2.6 folds of control on days 1, 7 and 28, respectively. Increased lung permeability was also observed following asbestos exposure with a maximal 2.05-fold increase in protein observed 7 days post exposure (Figure 4A).

The degree of pulmonary cytotoxicity elicited by SWCNT, CNF or asbestos was assessed by LDH activity in the BAL fluid recovered from mice. LDH levels were significantly elevated after exposure to CNF (120 μg/mouse; 1.8 fold vs control mice) on days 1 and 7 post exposure (Figure 4B). On day 28 post-CNF, LDH levels remained significantly (1.5 fold) elevated as compared to control mice. Similarly, the release of LDH in response to SWCNT and asbestos followed the same trends. Overall, SWCNT, CNF, and asbestos were all capable of inducing acute pulmonary cell damage with the potency as follows: SWCNT > CNF > asbestos.

### Oxidative stress in the lungs

**Figure 5:**
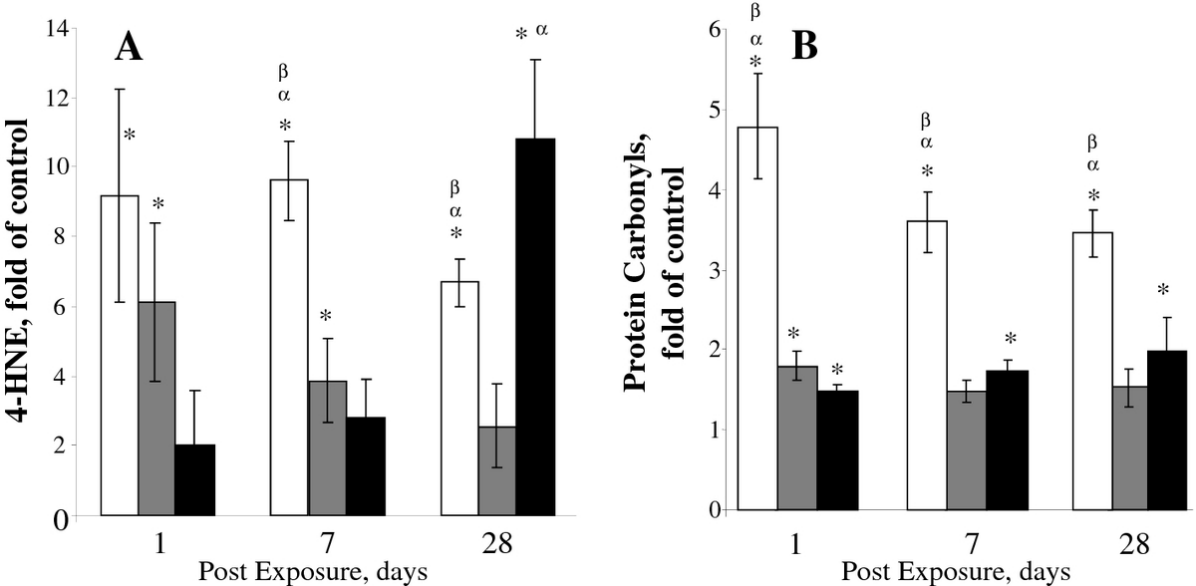
**Biomarkers of oxidative stress in the lung of C57BL/6 Mice following aspiration with CNF, Crocidolite Asbestos, or SWCNT**. A: 4-Hydroxynonenal; B: Protein Carbonyl. Mice were exposed via pharyngeal aspiration to the doses indicated. Open columns - exposure with SWCNT (40 μg/mouse); gray columns - exposure with CNF (120 μg/mouse); black columns - exposure with crocidolite asbestos (120 μg/mouse). Animals were sacrificed 1, 7, and 28 days post exposure. Means ± SE (*n *= 6 mice per group). *p < 0.05 vs. control (PBS) exposed mice; ^α^p < 0.05 vs. CNF exposed mice; ^β^p < 0.05 vs. asbestos exposed mice.

Oxidative damage assessed by levels of 4-hydroxynonenol (4-HNE) and oxidatively modified proteins (protein carbonyls) in the lungs of mice exposed to CNF, SWCNT, or asbestos is presented at Figure 5. The time course of 4-HNE accumulation in the lungs following CNF aspiration revealed a significant 6 and 4-fold increase (vs. control) after 1 and 7 days post exposure, respectively (Figure 5A). On day 28 post exposure, the levels of 4-HNE in the lungs of CNF exposed mice returned to control levels. As compared to CNF, SWCNT exposure (40 μg/mouse) induced a more pronounced accumulation of 4-HNE (9.5 and 10.0 fold vs control) on 1 and 7 days post exposure, persisting through day 28 post exposure (6.0 fold vs control). Asbestos exposure did not induce 4-HNE accumulation in the lungs on days 1 and 7 post exposure; however, a marked increase in amount of 4-HNE (11 fold vs control) was observed 28 days post exposure (Figure 5A).

Additionally, levels of oxidatively modified proteins, i.e. protein carbonyls, were evaluated in the lungs following SWCNT, CNF or asbestos exposure. SWCNT induced the most significant and sustained increase of protein carbonyls (4.8, 3.5, 3.5 fold vs control) found 1, 7, and 28 days post exposure, respectively. CNF and asbestos exposure (120 μg/mouse) elicited a steady 1.8-fold increase in protein carbonyls in the lungs of exposed mice (Figure 5B). Overall, SWCNT, CNF, and asbestos caused oxidation of proteins in the lungs with the magnitude of the oxidative damage as follows: SWCNT > CNF > asbestos.

### Cytokines

**Figure 6:**
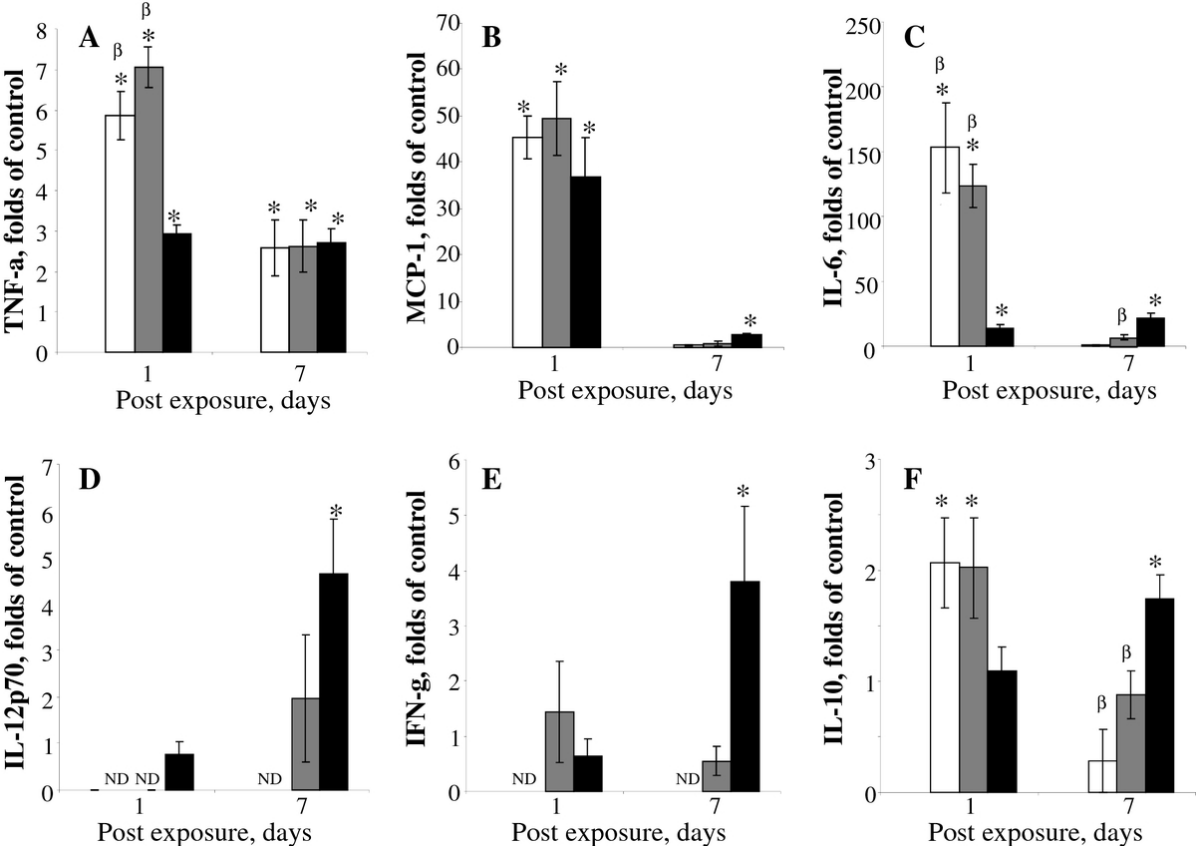
**Cytokine accumulation in bronchoaleolar lavage fluid of C57BL/6 mice following aspiration of CNF, Crocidolite Asbestos, or SWCNT**. A: TNF-α; B: MCP-1; C: IL-6; D: IL-12p70; E: IFN-γ; F: IL-10. Open columns - exposure with SWCNT (40 μg/mouse); gray columns - exposure with CNF (120 μg/mouse); black columns - exposure with crocidolite asbestos (120 μg/mouse). Mice were exposed via pharyngeal aspiration to the indicated doses. Animals were sacrificed 1, 7, and 28 days post exposure. Means ± SE (*n *= 6 mice per group).*p < 0.05 vs. control (PBS) exposed mice, ^β^p < 0.05 vs. mice exposed to asbestos.

Exposure to the studied NP resulted in accumulation of pro-inflammatory cytokines in the mouse lungs (Figure 6). The cytokine response peaked on day 1 post exposure to SWCNT or CNF (Figure 6). On day 7 post-SWCNT or CNF, levels of TNF-α and IL-10 remained significantly elevated (2.5 and 0.8 fold of control, respectively) as compared to controls (Figure 6A, F). In contrast, in asbestos-exposed mice the release of inflammatory cytokines peaked on day 7 post exposure (Figure 6).

### TGF-β1, collagen deposition and morphometry

**Figure 7:**
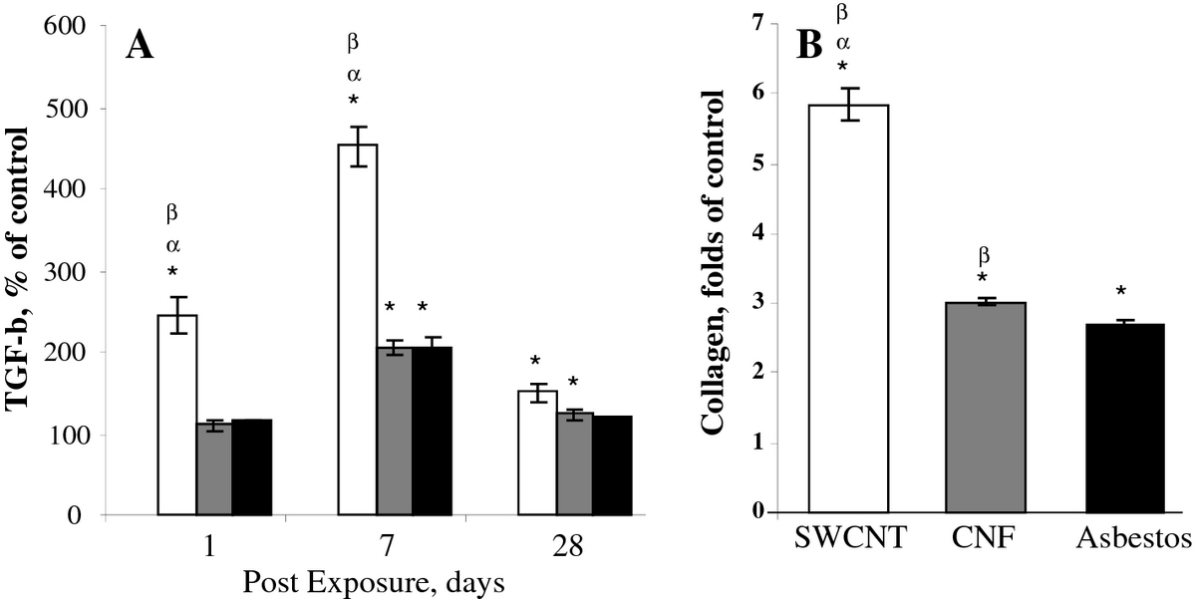
**Fibrogenic response as assessed by cytokine release in BAL fluid and collagen accumulation in the lung of C57BL/6 mice following CNF, Crocidolite Asbestos or SWCNT exposure**. A: TGF-β; B: Total collagen measured on day 28 post exposure. Open columns - exposure with SWCNT (40 μg/mouse); gray columns - exposure with CNF (120 μg/mouse); black columns - exposure with crocidolite asbestos (120 μg/mouse). Mice were exposed via pharyngeal aspiration to the indicated doses and animals were sacrificed 1, 7, and 28 days post exposure. Means ± SE (*n *= 6 mice per group). *p < 0.05 vs. control (PBS) exposed mice; ^α^p < 0.05 vs. mice exposed to CNF, ^β^p < 0.05 vs. mice exposed to asbestos.

**Figure 8:**
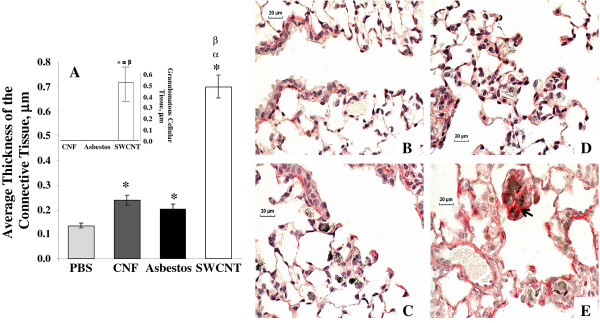
**Morphometric changes (A) and Sirius red-stained lung sections (B-E) from C57BL/6 mice 28 days following exposure to CNF, Crocidolite Asbestos or SWCNT**. A: Collagen fiber content determined as average thickness of connective tissue; Inset: Granulomatous cellular tissue; B: Control; C: CNF; D: Asbestos; E: SWCNT (Arrow indicate collagen accumulation). Mice were exposed via pharyngeal aspiration to 120 μg/mouse CNF or Asbestos, or 40 μg/mouse SWCNT. Animals were sacrificed 28 days post exposure. Means ± SE (*n *= 6 mice per group). *p < 0.05 vs. control (PBS) exposed mice, ^α^p < 0.05 vs. mice exposed to CNF, ^β^p < 0.05 vs. mice exposed to asbestos.

Significant elevation of TGF-β1 was found in BAL fluid of mice after SWCNT, CNF or asbestos exposure throughout the time course of the study (Figure 7A). Maximal TGF-β1 release in BAL fluid of mice was observed on day 7 post exposure. SWCNT induced the higher release of TGF-β1 on days 1, 7, and 28 post exposure (250, 450, and 125%, respectively, vs control), with the highest level found on day 7, while exposure to CNF or asbestos increased TGF-β1 up to maximum of 200% of control on day 7. A pronounced collagen accumulation was observed on day 28 post exposure in the lungs of mice treated with CNF or asbestos (3 or 2.8 -fold vs control, respectively) (Figure 7B). SWCNT exposure induced the most robust collagen buildup reaching 5.8 fold increase comparing to controls. Morphometric analysis of connective tissue stained with Sirius Red is given in Figure 8A. Deposition of collagen was observed in both granulomatous regions as well as in the areas distant from granulomas in the lung of mice exposed to SWCNT. This was established by conventional light microscopy of lung sections specifically stained with Sirius red (Figure 8E). The potency of alveolar interstitial fibrosis was as follows: SWCNT > CNF = asbestos (Figure 8).

### Histopathology results

**Figure 9:**
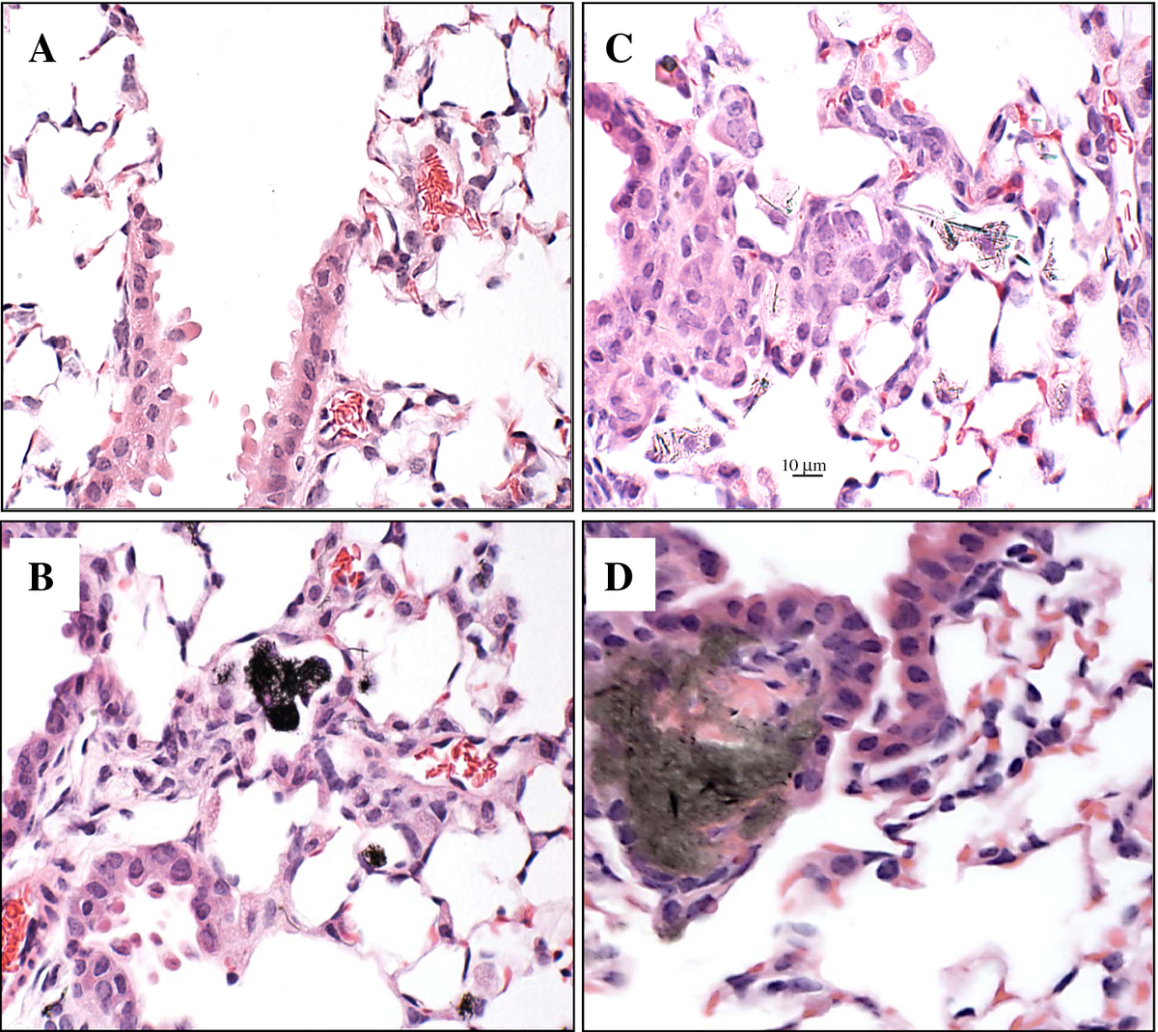
**Light micrographs of H&E-stained sections from lung of C57BL/6 mice 28 days following exposure to PBS (A), CNF (B), Crocidolite Asbestos (C) or SWCNT (D)**. Mice were exposed via pharyngeal aspiration to 120 μg/mouse CNF or Asbestos, or 40 μg/mouse SWCNT. Animals were sacrificed 28 days post exposure.

Histopathological changes in the lung of mice exposed to CNF, SWCNT or asbestos were evaluated by a board-certified veterinary pathologist. Representative micrographs from each group are shown in Figure 9. A chronic inflammatory reaction and fibrosis were observed 28 days following NP exposure (Figure 9B-D). Fibrous connective tissue was visualized in alveolar septa within the lungs of animals exposed to SWCNT, CNF or asbestos. In contrast to CNF and asbestos, SWCNT exposure revealed granulomatous inflammation, with discrete granulomas often surrounded by hypertrophied epithelioid macrophages associated with dense SWCNT agglomerates. Interfacing bundles of fibrous connective tissue were observed within discrete granulomas elicited by SWCNT agglomerates (Figure 9D).

### Proliferative response of splenic T cells from mice exposed to SWCNT, CNF, or asbestos

**Figure 10:**
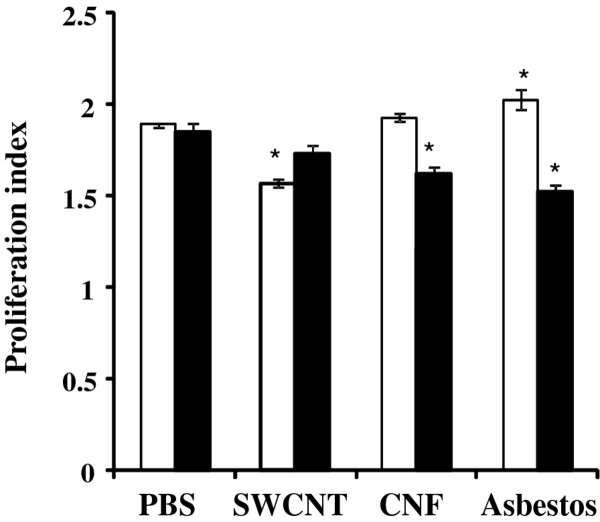
**Splenic T Cell Proliferation 7 and 28 days following pulmonary exposure to CNF, Crocidolite Asbestos or SWCNT**. Mice were exposed via pharyngeal aspiration to 120 μg/mouse CNF or Asbestos, or 40 μg/mouse SWCNT. Animals were sacrificed 7 and 28 days post exposure (white and black bars, respectively) and splenic T cell proliferation was evaluated. Means ± SE (*n *= 6 mice per group). *p < 0.05 vs. control (PBS) exposed mice.

To evaluate immune outcomes following exposure to respirable SWCNT, CNF or asbestos, we assessed the proliferative response of splenic T cells upon stimulation with concavalin A (T cell mitogen). No significant changes in proliferative response of splenic T cells from animals exposed to CNF were observed on day 7 post exposure, however a decreased proliferation was seen on day 28 post exposure. We found a decrease in proliferation of splenic T-cells obtained from SWCNT-exposed animals (~15% decrease vs control) on day 7 post exposure; however, it was returned to control values after 28 days. In contrast to CNF or SWCNT-treated mice, spleen T cells obtained from asbestos-treated animals showed an increased proliferative response on day 7 post exposure as compared to controls, while on day 28 we observed a decrease in responsiveness of the T cells to concavalin A (Figure 10).

### Correlation between effective surface area of nanomaterials administered and pulmonary outcomes

**Figure 11:**
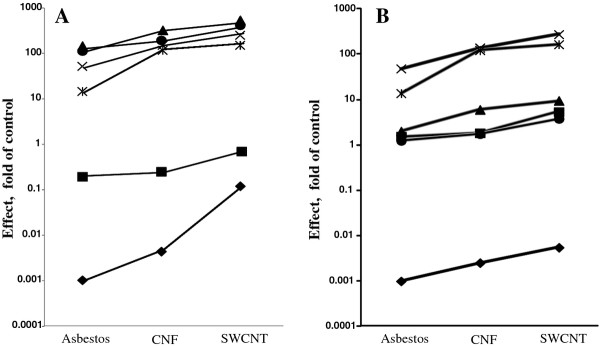
**Correlation between specific surface area or effective surface area of nanomaterials administered and pulmonary outcomes in the lung**. (A) Correlation between specific surface area of NM administered (as measured by BET) and pulmonary outcomes in the lung following exposure to Crocidolite Asbestos, CNF, or SWCNT on day 1 post exposure. Pearson's correlation coefficients were calculated for pairs of variables including NM dose (expressed as specific surface area of NM per mouse) and the relative values of the respective pulmonary outcomes: ♦ - NM dose, m^2^/mouse; ■ - alveolar wall thickness, day 28 post exposure (r = 0.995, p > 0.05); ▲ - 4-HNE, day1 post exposure (r = 0.837, p > 0.05; × - PMN counts, day1 post exposure (r = 0.908, p > 0.05); ✱ - IL-6, day1 post exposure (r = 0.733, p > 0.05); • - BAL protein, day1 post exposure (r = 0.979, p > 0.05). (B) Correlation between effective surface area of NM administered and pulmonary outcomes. Pearson's correlation coefficients were calculated for pairs of variables including NM dose (expressed as effective surface area of NM per mouse) and the relative values of the respective pulmonary outcomes: ♦ - NM dose, m^2^/mouse; ■ - alveolar wall thickness, day 28 post exposure (r = 0.963, p > 0.05); ▲ - 4-HNE, day1 post exposure (r = 0.974, p > 0.05; × - PMN counts, day1 post exposure (r = 0.997, p < 0.05); ✱ - IL-6, day1 post exposure (r = 0.905, p > 0.05); • - BAL protein, day1 post exposure (r = 0.997, p < 0.05).

Pearson's correlation coefficients were calculated for pairs of variables including NM dose, expressed as specific surface area (measured by BET, Figure 11A) or effective surface area (calculated as shown in Figure 2A-B) of NM per mouse (Figure 11B), and the relative values of the respective pulmonary outcomes. Several correlations with *effective *surface area were found to be statistically significant (P < 0.05), including PMN counts and total protein on day 1 post- exposure in BAL fluid (Figure 11B). In contrast, no statistically significant correlations were found between the *specific *surface area and pulmonary outcomes (Figure 11A).

## Discussion

Fibrous nanoparticles vary in length, shape, diameter, surface area, density, purity, content of transition metals, porosity and chirality. In aqueous milieu, carbonaceous NM tend to agglomerate and are rarely present as single entities [24]. In particular, aggregation/agglomeration of airborne CNF and CNT was previously reported in a series of field workplace studies [25-27]. In the current study, SWCNT appeared mainly as agglomerated structures composed of SWCNT bundled into ropes ranging 65-150 nm in diameter (Figure 1C). CNF were seen as agglomerates incorporating a few individual fibers with lengths varying from 5 to 30 μm, and widths within 80-160 nm range (Figure 1A). In contrast, asbestos fibers (2-30 μm in length and 0.16-0.8 μm in width) were mostly well dispersed with few detectable agglomerated structures (Figure 1B).

Pulmonary clearance of NM depends critically on their size and shape. Biopersistent, high aspect ratio fibers are recognized as a special hazard to the lungs. However, particle-like agglomerated structures of thinner CNT need to be distinguished from the fiber-like thick-walled and rigid assembly of nanofibers [24]. For fiber-like materials (asbestos, CNF, CNT), translocation to other tissues is determined by their dimensions, with the critical diameter < 0.4 μm and length < 10 μm [28]. The particle/agglomerate size as well as surface chemistry may be a significant factor affecting and/or limiting the recognition/engulfment of NM by alveolar macrophages [29,30]. Exposure of pulmonary cells (e.g. epithelial cells, macrophages, dendritic cells) to NM/fibers may ultimately lead to a broad variety of responses, ranging from cell damage/death (cytotoxicity) to engagement of intracellular signaling pathways facilitating the release of inflammatory mediators. Inflammation in the lung promotes myofibroblast recruitment and transformation, deposition of fibrin degradation products accelerating collagen production and pulmonary fibrosis. However, the prevailing mechanisms driving the fibrosis may be quite diverse for each particle/fiber. Previously, two distinct SWCNT particle morphologies were seen in SWCNT preparations employed for assessment of effects of respirable CNT: agglomerates and dispersed states [10]. Accordingly, two morphologically distinct responses were detected in the lungs as early as 7 days post exposure. Foci of granulomatous inflammation, including discrete granulomas often surrounded by hypertrophied epithelioid macrophages, were associated with deposition of SWCNT agglomerates. The SWCNT materials were clearly visualized within granulomatous lesions interfacing bundles of fibrous connective tissue. In lung regions distant from observed SWCNT agglomerates, morphological alterations were predominantly comprised of diffuse interstitial fibrosis with alveolar wall thickening. This interstitial fibrosis occurred at sites of deposition of more dispersed SWCNT structures [31]. Importantly, deposition of collagen and elastin was also observed in both granulomatous regions as well as in the alveolar walls [10]. In the current study, asbestos fibers did not form agglomerates in either of the aqueous preparation, (Figure 1B) or as deposited within the lungs of exposed animals (data not shown), while CNF fibers formed loosely packed agglomerates in both suspensions and in the lungs. The stiffness/rigidity of CNT or CNF could certainly have an impact on the agglomeration propensity and interactions of these materials with biological systems, and this has been the focus of several recent studies [32,33]. In the present study, we demonstrated that in an aqueous environment SWCNT and CNF tend to agglomerate, and such agglomerates would no longer obey the rules of non-agglomerating asbestos fibers. Therefore, interactions of agglomerated CNP with biological systems would be defined by the relative proportions of individual fibers vs. agglomerates present. In particular, no granuloma formation was found following exposure to fiber-like CNF particles/agglomerates or asbestos (Figure 9B, C). Therefore, granulomatous lesions formed after SWCNT exposure may be attributed to specific scaffolding features of SWCNT agglomerates. Here we demonstrate that SWCNT agglomerates induce granuloma formation, leading to morphological/structural isolation of SWCNT agglomerates within the lung, presumably making them less damaging to the surrounding pulmonary tissues. Agglomerated SWCNT in the lungs, once walled off by cuboidal cells, are less likely to cause acute inflammatory reactions. Thus, relatively rapid isolation of SWCNT aggregates/agglomerates within granulomas, not observed upon exposure to CNF or asbestos, may contribute to faster resolution of acute SWCNT-induced neutrophilic inflammation and pneumonia (Figure 3A). In addition, the potency for induction of alveolar interstitial fibrosis was as follows: SWCNT > CNF = asbestos (Figure 8A). Asbestos fibers are known to induce "frustrated phagocytosis" causing prolonged oxidative stress. However, no frustrated phagocytosis was seen following exposure of murine or human pulmonary phagocytes to SWCNT [30,34]. The relatively large calculated effective surface area of SWCNT (138 m^2^/g vs 21 and 8.3 m^2^/g for CNF and asbestos, respectively) aids to the adhesion of cell/tissue proteins to the surface of SWCNT [30,35]. In particular, the ability of SWCNT to serve as a scaffold is beneficial for the adhesion and proliferation of fibroblasts in the lungs and may be essential for their sturdy fibrogenic potential [36]. The unique surface structure of the SWCNT agglomerates and potential affinity to lipid and protein covalent binding and coating provides excellent environment facilitating growth and proliferation of fibroblasts [37]. It is noteworthy that CNF utilized in the current study share several physical properties with MWCNT, such as a relatively large diameter (as compared to SWCNT) which may contribute to higher stiffness, less "tangling" and lower agglomeration propensity for CNF as compared to SWCNT. Recently, MWCNT have been reported to elicit "asbestos-like" pathogenicity in rodent models, including lung injury and mesothelioma formation [32,33]. In the current study, we showed that the sub-acute inflammatory, immunologic and fibrogenic outcomes of pulmonary exposure to CNF are similar to asbestos. The carcinogenic potential of CNF as well as other relatively long-term outcomes is a matter for future investigations. Our data showed that agglomerated SWCNT and CNF do not behave as single, fibrous entities but rather as agglomerated particles, and subsequently do not follow the HARN paradigm. As demonstrated by Wang et al. [38], SWCNT directly stimulate fibroblast proliferation and collagen production in a cell culture system - in line with the known fact that lung fibroblasts like to grow upon SWCNT. This effect does not involve frustrated phagocytosis, as macrophages were not present in the system, but appears to involve the activation of matrix metalloproteinases (MMPs). It was recently shown that up-regulation of MMP-12 and cathespin K by SWCNT in co-culture of epithelial/mesenchymal lung cells and BAL macrophages was due to cell-type specific interactions [39]. The mechanisms of MMPs activation in response to SWCNT - known to cause the formation of irreversible interstitial fibrosis with airway alteration and changes in pulmonary functions found in mice [10,40,41] - resembled those that play a pivotal role in the pathogenesis of idiopathic fibrosis and obstructive airway disease in humans [42]."

Along with shape, size and structure, chemical composition of NM may also contribute to the inflammatory and toxic outcomes. SWCNT and CNF are produced predominantly by HiPco, chemical vapor deposition, laser ablation and arc discharge techniques involving utilization of various transition metal catalysts [43]. Catalytically competent metal-containing NM may synergistically enhance oxidative stress damaging the cells and tissues [34]. Kagan et al., [34] was one of the first to document - using EPR spectroscopy of ascorbate radicals as well as adducts with a spin-trap, 5,5-dimethyl-1-pyrroline-N-oxide (DMPO) - the production of hydroxyl radicals generated by iron admixtures in unpurified SWCNT as well as the suppression of these signals by an iron chelator, desferroxamine. SWCNT, CNF and asbestos employed in the current study were found to have 0.23, 1.4, and 18% iron, respectively. Iron content of unpurified SWCNT was previously implicated in enhanced oxidative stress, depletion of antioxidant reserves and accumulation of lipid peroxidation products after SWCNT exposures. Neither Fe-rich (unpurified) nor purified SWCNT were able to induce intracellular production of superoxide or nitric oxide by RAW 264.7 macrophages [34]. Instead, extracellularly generated highly reactive hydroxyl radicals, particularly in the presence of Fe-rich SWCNT, were reported to enhance the oxidative burst and cause oxidative stress via extracellular oxidation [34]. One should, however, keep in mind that SWCNT and CNF synthesized by high-pressure CO conversion (HipCo) or CVD methodology results in accumulation of mostly elemental iron embedded in the core crystalline structure of CNP and thereby not readily mobilized in water in ionic redox-active form. It is likely that uptake by professional phagocytes whereby SWCNT may be localized in acidic environments of phago-lysosomes will lead to the release of ionic iron, hence cause the toxic effects. This secondarily ionized form of iron may be involved in redox-cycling mechanisms and facilitate the development of oxidative stress. However, the significance of this oxidative stress for triggering non-specific peroxidation reactions is likely limited. In fact, mass-spectrometry based global lipidomics analysis of pulmonary lipid peroxidation after the inhalation exposure of mice to iron-rich SWCNT revealed a highly selective non-random pattern of phospholipid peroxidation whereby only anionic phospholipids cardiolipin, phosphatidylserine and phosphatidylinositol underwend oxidative modification [44]. These data are in sharp contrast with the expected random profile of peroxidation of the most abundant polyunsaturated species of phosphatidylcholine and phosphatidylethanolamine observed during non-selective transition metal-catalyzed peroxidation of phospholipids in tissues. In the current study, exposure to SWCNT, CNF and asbestos resulted in increased accumulation of biomarkers of oxidative stress, e.g. 4-hydroxynonenal (4-HNE) and protein carbonyls found in mouse lungs (Figure 5). Elevated levels of 4-HNE were found on days 1, 7 and 28 in SWCNT or CNF -treated animals, in contrast to the peak seen on day 28 in asbestos-treated mice (28 d). The most prominent induction of oxidative stress (4-HNE and protein carbonyls) occurred after SWCNT-exposure. We speculate that the relatively high surface area of carbonaceous NM facilitates the efficient interactions of catalytically active Fe with cellular components and pulmonary tissues; thereby explaining why SWCNT elicited the most pronounced oxidative stress, cell damage, granulomatous inflammation and fibrosis.

Inflammatory milieu in the lung launches a wide variety of signaling events engaging innate immunity and governing systemic/adaptive immune response. The pulmonary innate immune system provides rapid recognition of inhaled agents while orchestrating defensive responses. Exposure to airborne NM could engage pulmonary innate immunity at many levels. A number of recent publications have reported the effects of carbonaceous NM on the immune system [45-47]. It has been shown that splenic T cell dysfunction and impaired systemic immunity was associated with release of TGF-β and subsequent expression of IL-10 and PGE_2 _in the spleen [46]. Here, we observed that SWCNT elicited the most prominent release of TGF-β as compared to CNF and asbestos. Increased TGF-β found in BAL on day 7 post exposure to SWCNT was accompanied by slightly suppressed spleen T cell proliferation. At this time-point, release of TGF-β in response to CNF and asbestos was significantly lower as compared to that of SWCNT. Exposure to CNF or asbestos, in contrast to SWCNT, did not suppress T cell proliferation on day 7. Surprisingly, a slight stimulation of the spleen T cell responsiveness was observed on day 7 in animals exposed to asbestos (Figure 10). This stimulation may be partially attributable to a marked increase in IL-12 in the lung (Figure 6). However, 28 days post exposure, spleen T cell proliferation was suppressed in both CNF and asbestos treated animals, while pulmonary levels of TGF- β were not markedly changed. These data suggest that splenic T cell suppression at later time points (28 days) is not likely due to TGF- β release. CNF appears to have effects similar to asbestos causing a "delayed" immune suppression, which occurred when the acute inflammation was resolved. It has been reported that asbestos-related immune suppression followed 3 and 6 months after asbestos instillation [48]. One could expect that SWCNT with the highest surface area (138 m^2^/g) would elicit the strongest acute inflammation and release of TGF-β, as compared to CNF (21 m^2^/g) and asbestos (8.3 m^2^/g). At equivalent mass doses, CNF and asbestos are generally less capable of inducing TGF-β release in the lung; therefore, the peripheral tolerance/suppression observed is most likely driven by different mechanisms possibly involving suppressive antigen presenting cells (APC) [46] and regulatory T cell induction.

In order to address whether specific surface area and/or particle number derived from toxicological studies is useful as a dose metric for hazard identification and risk assessment, we attempted to correlate the inflammatory (PMN) responses observed in the lungs of mice to either specific surface area (measured by BET) or number of particles/agglomerates in the given amount (mass) of NM (Figure 11A). The dose of SWCNT given to animals (40 μg/mouse) was equivalent to the specific surface area of SWCNT of 4.16 × 10^-2 ^m^2^/mouse, while CNF and asbestos doses (120 μg/mouse) were equal to 5.4 × 10^-3 ^m^2^/mouse and 9.6 × 10^-4 ^m^2^/mouse, respectively. The data presented in Figure 11 indicate that although the specific surface area of SWCNT (measured by BET) given to mice was 43 times higher as compared to asbestos, PMN counts in BAL fluid of mice exposed to SWCNT were only 5.7 fold higher (1 day post exposure). Moreover, on day 7 post exposure PMN counts in animals exposed to asbestos were 12.2 fold higher as compared to SWCNT. Accordingly, particle number alone does not seem to be a reliable factor in dose metrics for assessment of NM exposure outcomes. At the same concentration, SWCNT and CNF suspensions had a much lower number of particles/structures due to agglomeration as compared to non-agglomerated asbestos (Figure 1D); however, the neutrophilic infiltration in the lung of animals exposed to SWCNT and CNF was greater as compared to asbestos-exposed mice (24 h post exposure). These data suggest that specific surface area (measured by BET) or particle/agglomerate numbers do not provide a reliable basis for predicting biological outcomes of exposure to carbonaceous NM and is therefore not an efficient dose metric for the assessment of pulmonary outcomes in response to agglomerating fibrous NM.

Indeed, PMN counts in BAL fluid of mice exposed to SWCNT or CNF were increased by ~5.7 and ~2.8 fold, respectively, as compared to asbestos. Of note, the calculated effective surface areas of SWCNT and CNF agglomerates delivered to the lung were ~5.8- and ~2.6- fold higher (vs. asbestos, respectively, day 1 post exposure). Correlations between various pulmonary outcomes and effective surface area of NM administered to the animals are presented on Figure 11. Protein levels in BAL fluid of mice exposed to NM (day 1 post exposure) were well correlated with the calculated effective surface area of particle agglomerates given to animals: Pearson's correlation coefficient was 0.997, p < 0.05. Additionally, our data suggests that the high aspect ratio nanoparticle (HARN) paradigm [49] is not fully applicable for the assessment of the hazardous effects of carbonaceous fibrous NM. Therefore, in addition to mass dose, the effective surface area of NM structures should be experimentally determined by detailed analysis of NM agglomerates. Effective surface area of NP agglomerates may be useful as a predictive dose metric of pulmonary toxicity - acute inflammation, pulmonary damage and fibrosis - induced by SWCNT or CNF and could thus be utilized for health hazard and risk assessment of fibrous carbonaceous NM.

## Conclusions

1. CNF, SWCNT and asbestos cause inflammation, pulmonary damage and fibrosis in the lung of mice with the following mass-based potency: SWCNT > CNF ≥ asbestos. Early and robust fibrosis elicited by SWCNT may be partially attributed to scaffolding properties of SWCNT.

2. Exposure to SWCNT, CNF and asbestos resulted in oxidative stress in the lung. Despite the higher iron content in asbestos, SWCNT and CNF caused more severe oxidative stress/damage as compared to asbestos.

3. SWCNT, CNF and asbestos were able to modulate local and systemic immunity upon pulmonary exposure. At equivalent mass doses, CNF and asbestos are generally less capable of inducing suppressive TGF-β1 release in the lung; therefore, the peripheral tolerance/suppression observed is most likely driven by mechanisms involving APC such as DCs.

4. It is questionable if agglomerated SWCNT and CNF structures can be considered conventional fibers; hence, their effects may not be readily understood according to the HARN paradigm.

5. Specific surface area and particle number do not necessarily predict biological/toxicological responses to carbonaceous fibrous NM in living organisms, and therefore are not - according to the present data - the most appropriate dose metric for agglomerating NM.

6. Values of effective calculated surface area of agglomerated NM could be useful for prediction of biologic responses e.g. pulmonary inflammation, damage and fibrosis.

## Materials and methods

### Animals

Specific pathogen-free adult female C57BL/6 mice (8-10 wk) were supplied by Jackson Lab (Bar Harbor, ME) and weighed 20.0 ± 1.9 g when used. Animals were housed one mouse per cage receiving HEPA filtered air in the AAALAC-accredited NIOSH animal facilities. All animals were acclimated in the animal facility under controlled temperature and humidity for one week prior to use. Beta Chips (Northeastern Products Corp., Warrensburg, NY) were used for beddings and changed weekly. Animals were supplied with water and certified chow 7913 (Harlan Teklad, Indianapolis, IN) ad libitum, in accordance with guidelines and policy set forth by the Institute of Laboratory Animals Resources, National Research Council. All experimental procedures were conducted in accordance with a protocol approved by the NIOSH Institutional Animal Care and Use Committee.

### Experimental design

A suspension of SWCNT (40 μg/mouse), CNF (120 μg/mouse) or asbestos (120 μg/mouse) was used for single pharyngeal aspiration of C57BL/6 mice, while the corresponding control mice were administered sterile Ca^+2 ^+ Mg^+2^-free phosphate-buffered saline (PBS) vehicle. Mice were sacrificed on days 1, 7 and 28 following exposure. All experiments were repeated at least three times. Inflammation was evaluated by total cell counts, cell differentials, and accumulation of cytokines in the bronchoalveolar lavage (BAL) fluid. Pulmonary toxicity was assessed by elevation of LDH activity in acellular BAL fluid. Fibrogenic responses to exposed materials were assessed by alveolar wall thickness morphometry and collagen deposition. For each group six animals were used to do BAL analysis, histopathology evaluation, oxidative stress markers, and lung collagen measurement.

### Particles

CNF were obtained from Pyrograf Products, Inc. Carbon nanofibers were vapor grown (PR-24, LHT grade) and heat treated (up to 3000°C) to graphitize chemically vapor deposited carbon present on the surface of the pyrograf and to remove iron catalyst. SWCNT (Unidym Inc, Sunnyvale, CA) were manufactured using the high pressure CO disproportionation process (HiPco™) and purified with acid treatment to remove catalytic metal contaminants [50]. An UICC standard crocidolite asbestos was utilized for comparison of fiber effects. Total elemental carbon and trace metal analysis was performed by the Chemical Exposure and Monitoring Branch (DART/NIOSH, Cincinnati, OH). Elemental carbon was assessed according to the NIOSH Manual of Analytical Methods (NMAM) [51], while trace metal were analyzed by nitric acid dissolution and inductively coupled plasma-atomic emission spectrometry (ICP-AES) following NMAM method 7300 for trace metals. Raman spectroscopy, near-infrared (NIR) spectroscopy, and thermo-gravimetric analysis (TGA) were used for purity assessment of HiPco™ SWCNT. Specific surface area was measured at -196°C by the nitrogen absorption-desorption technique (Brunauer Emmet Teller method, BET) using a SA3100 Surface Area and Pore Size Analyzer (Beckman Coulter Inc, Fullerton, CA), and diameter was measured by TEM. For all size distribution measurements and animal exposures, divalent ion free PBS was utilized as a particle dispersion medium. Prior to animal exposure or size measurements, particles were ultrasonicated (30s × 3 cycles) for improved dispersion of nanoparticles using Vibra Cell (Sonics and Materials Inc., CT, USA) probe sonicator operating at 20 kHz (65% power).

### Particle imaging and size measurements

Images of NP suspensions were obtained by field emission scanning electron microscopy. The size distribution of samples, including agglomerate measurements, were performed as previously described by Wang et al. [52]. In brief, the particles deposited on polycarbonate filter were viewed under a field emission scanning electron microscope (model S-4800; Hitachi, Tokyo, Japan) at 400 and 30,000 magnifications. The average length and width of the structures in each sample were determined by analysis of a minimum of 300 particles. Number concentrations of structures were estimated by analyzing 10 fields of view for every particle type/suspension. Agglomerates were counted as a single entity.

### Particulate instillation

Mouse pharyngeal aspiration was used for particulate administration. Briefly, after anesthetization with a mixture of ketamine and xylazine (62.5 and 2.5 mg/kg subcutaneous in the abdominal area), the mouse was placed on a board in a near vertical position and the animal's tongue extended with lined forceps. A suspension (approximately 50 μl) of SWCNT (40 μg/mouse), CNF (120 μg/mouse), or asbestos (120 μg/mouse) prepared in divalent ion free PBS was placed posterior on the throat and the tongue which was held until the suspension was aspirated into the lungs. Control mice were administered sterile Ca^+2 ^+ Mg^+2^-free phosphate-buffered saline (PBS) vehicle. The mice revived unassisted after approximately 30-40 min. All mice in PBS, SWCNT, CNF, and asbestos groups survived this exposure procedure. This technique provided good distribution of particles widely disseminated in a peri-bronchiolar pattern within the alveolar region as was detected by histopathology [53]. Animals treated with the particulates and PBS recovered easily after anesthesia with no behavioral or negative health outcomes. Mice were sacrificed on days 1, 7, and 28 days following the exposure.

### Estimation of effective surface area from geometric analysis of SWCNT and CNF's

The theoretical effective surface area (ESA) was estimated based on the geometrical analysis of carbon nanotubes and nanofibers, using the CNT surface area models developed previously [54]. Assuming that both CNT's and CNF's are generated from the base material graphene, the ESA of SWCNT bundle can be estimated as the product of specific surface area (SSA) of graphene (in m^2^/g) and the effective surface area of a N layered SWCNT bundle. The SSA of a graphene was estimated as 1315 m^2^/g. The effective surface area(N_eq-ssa_) of N layered (N_L_) SWCNT bundle can be obtained using the ratio between the number of individual SWCNT with a total accessible surface area equal to that of a bundle made up of N SWCNT and the total number of SWCNT's (N) in a bundle. The parameters N_L_, N_eq-ssa _and N of a bundle can be estimated as follows:

(1) $${\text{N}}_{\text{L}}=0.\text{5}\times \left(\text{diameter of the bundle}/\text{diameter of SWCNT}\right)-0.\text{5}$$

(2) $${\text{N}}_{\text{eq}-\text{ssa}}=\left(\text{3}\times {\text{N}}_{\text{L}}\right)+\text{1}$$

(3) $$\text{N}=\text{6}.\text{3791}\times {{\text{N}}_{\text{L}}}^{\left(\text{1}..\text{6646}\right)}$$

(4) $$\text{ESA}\left(\text{bundle}\right)=\text{SSA of graphene}\times \left({\text{N}}_{\text{eq}-\text{ssa}}/\text{N}\right)=\text{1315}\times \left({\text{N}}_{\text{eq}-\text{ssa}}/\text{N}\right)$$

In order to estimate the ESA of CNFs, we assumed the concentric graphitic sheets of CNFs are approximately similar to the model of MWNT proposed by Peigney et al. [54]. To calculate thickness of the walls and number of carbon layers of CNF in our study, we determined the average diameter of the hollow core (d_HC_) and outer diameters (d_OD_) of the CNF based on the SEM images taken (Figure 1A). Assuming that the inter-wall distance between any two carbon layers is 0.34 nm, number of layers (n-layers) in a CNF and ESA of CNF can be estimated using the following equations.

(5) $$\text{n}-\text{layers}=\left(\left({\text{d}}_{\text{OD}}\right)-\left({\text{d}}_{\text{HC}}\right)\right)/\left(2\times 0.34\right)$$

(6) $$\text{ESA}\left(\text{CNF}\right)=\left(\text{SSA of graphene}\times \left({\text{d}}_{\text{OD}}\right)\right)/\left(\left(\text{n}-\text{layers }\times \left({\text{d}}_{\text{OD}}\right)\right)\right)-\left(\text{2}\times 0.\text{34 }\times \left(\left(\overset{n-1}{\underset{i=1}{\sum }}i\right)\right)\right)$$

### Obtaining bronchoalveolar lavage (BAL) from mice

Mice were weighed and sacrificed with intraperitoneal injection of sodium pentobarbital (> 100 mg/kg) and exsanguinated. The trachea was cannulated with a blunted 22 gauge needle, and BAL was performed using cold sterile PBS at a volume of 0.9 ml for first lavage (kept separate) and 1.0 ml for subsequent lavages. Approximately 5 ml of BAL fluid per mouse was collected in sterile centrifuge tubes. Pooled BAL cells for each individual mouse were washed in PBS by alternate centrifugation (800 ×*g *for 10 min at 4°C) and resuspension. Cell-free first fraction BAL aliquots were stored at 4°C for LDH assays while the remainder was frozen at -80°C until processed.

### BAL cell counting and differentials

The degree of inflammatory response induced by pharyngeally aspirated SWCNT, CNF or asbestos was estimated by quantitating total cells, alveolar macrophages (AMs), and polymorphonuclear leukocytes (PMNs) recovered by BAL. Cell counts were performed using an electronic cell counter equipped with a cell sizing attachment (Coulter model Multisizer II with a 256 C channelizer, Coulter Electronics, Hialeah, FL). Alveolar macrophages (AM), and PMNs, were identified by their characteristic cell shape in cytospin preparations stained with Diffquick (Fisher Scientific, Pittsburgh, PA), and differential counts of BAL cells were carried out. Three hundred cells per slide were counted.

### Total protein and lactate dehydrogenase (LDH) activity in the BAL fluid

Measurement of total protein in the BAL fluid was performed by a modified Bradford assay according to the manufacturer's instructions (BioRad, Hercules, CA) with bovine serum albumin as a standard. The activity of LDH was assayed spectrophotometrically by monitoring the reduction of nicotinamide adenine dinucleotide at 340 nm in the presence of lactate using Lactate Dehydrogenase Reagent Set (Pointe Scientific, Inc., Lincoln Park, MI).

### Lung lavage fluid cytokine analysis

Levels of cytokines were assayed in the acellular BAL fluid following SWCNT, CNF or asbestos aspiration. The concentrations of TNF-α, MCP-1, IL-12, IL-6, IL-10 and IFN-γ (sensitivity of assay is 5-7.3 pg/ml) were determined using the BD™ Cytometric Bead Array, Mouse Inflammation kit (BD Biosciences, San Diego, CA). The concentration of active TGF-β1, (sensitivity of assay is < 15.6 pg/ml) was determined using an ELISA kit (Biosource International Inc., Camarillo, CA).

### Preparation of lung homogenates

The whole mouse lungs were separated from other tissues and weighed before being homogenized with a tissue tearer (model 985-370, Biospec Products Inc., Racine, WI) in PBS (pH 7.4) for 2 min. The homogenate suspension was frozen at -80°C until processed.

### Evaluation of biomarkers of oxidative stress in the lung

Oxidative damage to the lung following exposure to CNF, SWCNT, or asbestos was evaluated by the presence of 4-hydroxynonenol (4-HNE) and protein carbonyl formation. 4-HNE, a byproduct of lipid peroxidation, was measured in lung homogenates by ELISA using the OxiSelect HNE-His adduct kit (Cell Biolabs, Inc, San Diego, CA). The quantity of oxidatively modified proteins as assessed by measurement of protein carbonyls in lung homogenates was determined using the Biocell PC ELISA kit (Northwest Life Science Specialties). Sensitivity of the assay is < 0.1 nmol/mg protein.

### Lung preparation for microscopic evaluation

Preservation of the lung was achieved by vascular perfusion with a glutaraldehyde (2%), formaldehyde (1%), and tannic acid (1%) fixative with sucrose as an osmotic agent [55]. This method of fixation was chosen to prevent possible disturbances of the airspace distribution of deposited materials while maintaining physiological inflation levels comparable to that of the end expiratory volume. This was performed using protocols previously employed to study pulmonary effects of SWCNT [10]. Briefly, animals were deeply anesthetized with an overdose of sodium pentobarbital by subcutaneous injection in the abdomen, the trachea was cannulated, and laparotomy was performed. Mice were then sacrificed by exsanguination. The pulmonary artery was cannulated via the ventricle and an outflow cannula inserted into the left atrium. In quick succession, the tracheal cannula was connected to a 5 cm H_2_O pressure source, and clearing solution (saline with 100 U/ml heparin, 350 mosM sucrose) was perfused to clear blood from the lungs. The perfusate was then switched to the fixative. Coronal sections were cut from the lungs. The lungs were embedded in paraffin and sectioned at a thickness of 5 μm with an HM 320 rotary microtome (Carl Zeiss, Thornwood, NY). Lung sections for histopathological evaluation were stained with hematoxylin and eosin and examined by a board certified veterinary pathologist for morphological alterations.

### Lung collagen measurements

Total lung collagen content was determined by quantifying total soluble collagen using the Sircol Collagen Assay kit (Accurate Chemical and Scientific Corporation, Westbury, NY). Briefly, whole lungs were homogenized in 0.7 ml of 0.5 M acetic acid containing pepsin (Accurate Chemical and Scientific Corporation, Westbury, NY) with 1:10 ratio of pepsin: tissue wet weight. Each sample was stirred vigorously for 24 h at 4°C, centrifuged, and 200 μl of supernatant was assayed according to the manufacturer's instructions.

### Morphometric evaluation Sirius red staining of lung sections

The distributions of type I and III collagen in the lung tissue were determined by morphometric evaluation of the Sirius red-stained sections. Briefly, paraffin lung sections (5-μm thick) were deparaffinized and dehydrated. To identify collagen fibers under the microscope, the sections were stained with F3BA/picric acid for 1-2 h, washed with 0.01 N HCl for 1 min, and counterstained with Mayer's hematoxylin for 2 min. The slides were then dehydrated and mounted with a coverslip [56]. Type I and III collagen stained by Sirius red was visualized, and six randomly selected areas were scored under polarized microscopy using image analysis. With this morphometric method, the average thickness of Sirius red-positive connective tissues in the alveolar wall was quantitatively measured. Volume and surface density were measured using standard morphometric analyses of points and intercept counting [57]. Average thickness of the Sirius red-positive connective tissues of the alveolar wall was computed from two times the ratio of volume density of points to the surface density of the alveolar wall.

### Spleen harvest and cell isolation

Spleens from C57BL/6 mice following exposure to PBS, CNF, SWCNT, or asbestos were obtained on day 7 and 28 post exposure. Spleens were aseptically harvested then ground and the suspension was filtered through a cell strainer. Isolated splenocytes were then centrifuged and red blood cells were lysed utilizing red blood cell lysing buffer (Sigma).

### Splenocyte proliferation, ex vivo

Splenocytes were obtained from exposed (120 μg/mouse CNF, asbestos or 40 μg/mouse SWCNT) or nonexposed C57BL/6 mice (day 7 and 28 post exposure). Cells were labeled with 5-(and-6)-carboxyfluorescein diacetate at 5 μM concentration for 5 min (Invitrogen, Carlsbad, CA), counted using a hemacytometer, diluted in complete medium (1 × 10^6 ^cells/mL), and stimulated with 5 μg/mL concavalin A (Sigma, St. Louis, MO) for 4 days in 24-well plates in triplicates. The proliferation response was measured using flow cytometry (BD FACSCalibur instrument, BD, NJ). Dead cells were excluded from the assay with propidium iodide staining preceding the flow cytometry. The background fluorescence readings were subtracted during the analysis. Proliferation index is the average number of cell divisions that the responding T cells underwent. Only responding T cells are reflected in the proliferation index. The proliferation indices were calculated from flow cytometry data using the Flowjo software package (Tree Star Inc., Ashland, OR).

### Statistics

Treatment related differences were evaluated using two-way ANOVA, followed by pair wise comparison using the Student-Newman-Keuls tests, as appropriate. Pearson's correlation coefficients (r) were calculated for pairs of variables including NP effective surface area and relative values of PMN, protein, 4-HNE, IL-6 and alveolar wall thickness. The degrees of freedom (df) for correlation calculations were considered 1. Statistical significance was considered at p < 0.05. Data are presented as Mean ± SE.

## Abbreviations

CNT: Carbon nanotubes; CNF: Carbon nanofibers; SWCNT: Single-walled carbon nanotubes; MWCNT: Multi-walled carbon nanotubes; NM: Nanomaterials; MΦ: Macrophage; ROS: Reactive oxygen species; RNS: Reactive nitrogen species; HARN: High aspect ratio nanoparticle; PBS: Phosphate buffered saline; HiPco: High pressure CO disproportionation process; NMAM: NIOSH manual of analytical methods; ICP-AES: Inductively coupled plasma-atomic emission spectrometry; NIR: Near-infrared spectroscopy; TGA: Thermo-gravimetric analysis; SSA: Specific surface area; ESA: Effective surface area; BAL: Bronchoalveolar lavage; AMs: Alveolar macrophages; PMNs: Polymorphonuclear leukocytes; LDH: Lactate dehydrogenase; 4-HNE: 4-hydroxynonenol; NP: Nanoparticles; APC: Antigen presenting cell.

## Competing interests

The authors declare that they have no competing interests.

## Authors' contributions

AM, EK, and AT completed the experiments, analyzed data, and prepared the manuscript. NY evaluated and calculated the theoretical surface area of the NPs by geometrical analysis. RM completed morphometric evaluation of mouse lung sections. SHY completed and analyzed cytokine levels in BALF. VK and BF assisted in interpreting the data and drafting the manuscript. AS was involved in experimental design, coordinating the study, interpreting the data, and writing the manuscript. All authors read and approved the final manuscript.
